# The Diagnosis of Metastatic Axillary Lymph Nodes of Breast Cancer By Diffusion Weighted Imaging: a meta-analysis and systematic review

**DOI:** 10.1186/s12957-016-0906-5

**Published:** 2016-06-02

**Authors:** Wei Fan Sui, Xiang Chen, Zhen Kun Peng, Jing Ye, Jing Tao Wu

**Affiliations:** Radiology Department, Subei People’s Hospital of Jiangsu Province, No.98 of the Nantong West Road, Yang Zhou, Jiang Su Province China

**Keywords:** Diffusion-weighted imaging, Apparent diffusion coefficient, Breast cancer, Axillary lymph node metastases, Meta-analysis and systematic review

## Abstract

**Background:**

The purpose of this meta-analysis was to evaluate the clinical significance of diffusion-weighted imaging in assessing the status of axillary lymph nodes in patients with breast cancer.

**Methods:**

We searched the PubMed, Cochrane, and EMBASE databases, selected studies by inclusion and exclusion criteria, and assessed the quality of selected studies. We explored the source of heterogeneity; calculated sensitivity, specificity, positive and negative likelihood ratios, and pretest probability. A summary receiver operating characteristic curve was performed. Student’s *t* test was used to compare the different mean apparent diffusion coefficient values of different status lymph nodes.

**Results:**

In selected 10 studies, a total of 801 patients and 2305 lymph nodes were included following inclusion criteria. All scores of the quality assessment of the included studies were greater than or equal to 10 points. The sensitivity was 0.89 (95 % CI 0.79–0.95), the specificity was 0.83 (95 % CI 0.71–0.91), the positive and negative likelihood ratios were 3.86 (95 % CI 2.75–5.41) and 0.17 (95 % CI 0.09–0.32), the pretest probabilities were 53 and 54 %, the area under the curve were 0.93 (95 % CI 0.90–0.95), respectively. The mean apparent diffusion coefficient value of metastatic lymph nodes was significantly lower than that of nonmetastatic axillary lymph nodes.

**Conclusions:**

Diffusion-weighted imaging is a promising tool to discriminate between metastatic and nonmetastatic axillary lymph nodes. Combined with the mean apparent diffusion coefficient value, it can quantitatively diagnose lymph node metastases. Conducting large-scale, high-quality researches can improve the clinical significance of diffusion-weighted imaging to distinguish metastatic and nonmetastatic axillary lymph nodes in patients with breast cancer and provide the evidence to assess the status of axillary lymph nodes.

## Background

Evaluating the status of axillary lymph nodes (ALNs) is crucial in staging, deciding the treatment planning, and predicting the long-term survival in breast cancer [1–3]. Biopsy is recognized as the gold standard for assessing ALNs. However, the drawbacks of biopsy are high false negative ratio result from sample errors and its invasiveness [4]. The imaging modalities for assessing the ALNs are rapidly evolving. Ultrasound (US) is applied widely for its convincing and dynamic observation. However, the sensitivity and specificity of ultrasound for lymph node metastasis were unreliable and controversial [5, 6]. Owing to radiation and relative lower diagnostic accuracy, computer tomography (CT) is limited in clinic [7]. Positron emission tomography (PET) and positron emission tomography/computed tomography (PET/CT) can reflect metabolism of glycolytic activity. Undoubtedly, they have shown the higher diagnostic significance in assessing distant metastases and regional metastatic ALNs [8], but their high radiation and expensive fee keep the common people away. Ultrasmall super paramagnetic iron oxide (USPIO) is the same. Its satisfactory performance was extinguished by drawbacks of time-consuming, underlying risk and forbidden in clinical practice [9].

Magnetic resonance imaging (MRI) is developing with an unimaginable speed; over the past years, it has been used to evaluate ALNs [10]. MRI can detect deep and contralateral lymph nodes. Its sensitivity and specificity for metastatic ALNs were higher than US and CT [11]. Diffusion-weighted imaging (DWI) as an advanced technology of MRI with its superior ability to apparent the diffusion of water molecules freely in tissues [12] has also been useful for diagnosing lymph nodes in the axillary and other sites. Apparent diffusion coefficient (ADC) can quantify the diffusion of water molecules; ADC value and ADC ratio were applied to distinguish metastatic from nonmetastatic lymph node widespread [13].

The role of DWI in diagnosing the metastatic ALNs can be found in several published studies; however, the existing results in these studies were enormously varied, and a comprehensive systematic review would be useful to synthesize the current available information. The aim of this meta-analysis was to assess the clinical value of DWI in detecting ALN metastases in patients with breast cancer.

## Methods

### Literature search

We searched studies about the diffusion-weighted imaging in diagnosing lymph node metastases in the PubMed, EMBASE, and Cochrane library until May 2015 following the key words “diffusion-weighted,” “diffusion weighted imaging,” “diffusion weighted magnetic resonance imaging,” “DWI,” “lymph nodes,” “axillary,” “breast cancer,” “breast carcinoma,” “ductal cancer,” “tubular cancer,” and “medullar cancer”.

### Inclusion and exclusion criteria

We reviewed studies for the following inclusion criteria: (1) studies were published in English, (2) DWI was performed in detecting ALNs with breast cancer, (3) histopathological results were used as the reference standard, and (4) the true positive (TP), false positive (FP), true negative (TN), and false negative (FN) values can be calculated with sufficient data. We excluded the studies with the following criteria: (1) lack the explanations of DWI-detected ALNs with breast cancer; (2) without histopathological reference standard; (3) insufficient data to get the TP, FP, TN, and FN values; (4) experimental subject was an animal and ex vivo; (5) the type of study was review, case report, letter to editor, and meta-analysis; and (6) unable to get the full text.

### Data extraction and quality assessment

We extracted the following items in each study: author, nation, publication year, sample size, *b* value, field strength, the cutoff of ADC value, the mean ADC value of the metastatic and nonmetastatic ALNs, study design, and TP, FP, TN, and FN values.

Assessing studies through QUADAS [14], the total score of the included studies must be greater than 10 points or equal to 10 points.

### Statistical analysis

We applied Meta-DiSc version 1.4, Stata 12.0, SPSS 19.0, to analyze data.

In order to assess the clinical significance of DWI, we calculated sensitivity, specificity, positive and negative likelihood ratios (PLR, NLR), and pretest probability and performed a summary receiver operating characteristic (SROC) curve based on the collected studies of TP, FP, TN, and FN values.

Heterogeneity can affect the accuracy of estimation, and *I*^2^ index was used to evaluate heterogeneity. There was existing heterogeneity when *P* value was less than 0.05 and/or *I*^2^ value was over 50 % [15]. In diagnostic meta-analysis, the threshold effect was considered as an important source of heterogeneity. We used Spearman correlation coefficient in Meta-DiSc version 1.4 to check it. If threshold effect existed in our analysis, we summarized receiver operating characteristic (ROC) curve and calculated the area under the curve (AUC) directly. If no threshold effect existed in our analysis, after calculating the SROC and AUC, we also used meta-regression and subgroup analysis to explore the source of heterogeneity in Meta-DiSc version 1.4. We used Deeks’ funnel plot in Stata 12.0 to assess publication bias [16].

Owing to the different mean ADC values in metastatic and nonmetastatic ALNs, we used Student’s *t* test in SPSS 19.0 to compare the different mean ADC values of different status ALNs.

## Results

### Selection of studies

**Fig. 1:**
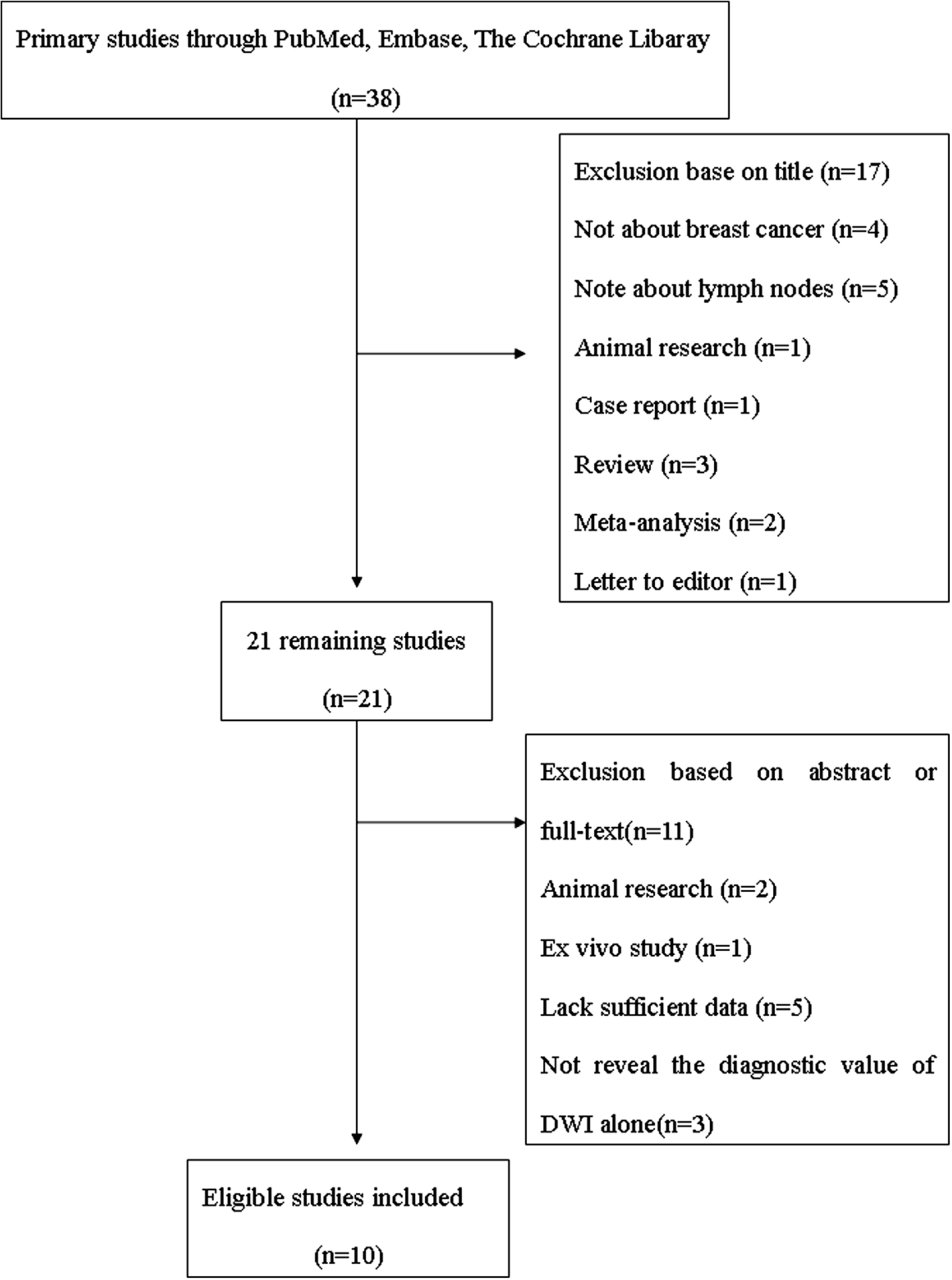
Flow chart: selection process of the studies

We total yielded 38 primary studies after searching the PubMed, EMBASE, and Cochrane library. We excluded 17 studies after reading the title. From the remaining 21 studies, we excluded 11 studies after reading abstract or full text and included 10 studies [13, 17–25]. The detail of selection can be shown in Fig. 1.

### Data extraction and study assessment

This study included a total of 801 patients and 2305 ALNs of the included 10 studies in this meta-analysis. The detail characteristic data can be seen in Table 1. All the selected studies were conducted with the QUADAS test, and their scores were greater than 10 points or equal to 10 points (Table 2).

**Table 1:** Characteristics of inclusion articles

Author	Year	Nation	Design	No. of patients and nodes	*b* value (s/mm^2^)	Field strength (T)	ADC cutoff (×10^−3^mm/s^2^)	Mean ADC of metastases (×10^−3^mm/s^2^)	Mean ADC of benign (×10^−3^mm/s^2^)	Coil	Reference standard	tp	fp	fn	tn
Nakai	2011	Japan	NG	16, 216	0, 800	1.5	NG	1.04	1.22	Cardiac surface	Histopathologically	30	4	6	176
Kamitani	2012	Japan	Retro	108, 110	0, 1000	1.5	1.05	1.08	0.92	Body	Histopathologically	14	11	12	73
Fornasa	2013	Italy	Prosp	43, 43	0, 800	1.5	1.09	0.88	1.4	4-channel phased array	Histopathologically	18	2	1	22
He	2013	China	NG	136, 1242	500	1.5	1.68	1.37	1.77	8-channel phased array breast	Biopsy or surgical resection	138	498	4	602
					800		1.35	1.18	1.55			134	376	8	724
Luo	2013	China	NG	36, 79	0, 800	1.5	0.889	0.79	1.04	8-channel phased array breast	Histopathologically	37	6	8	28
Chung	2013	South Korea	NG	110, 110	0, 1000	1.5 3.0	0.9	0.69	1.04	4-channel phased array breast	Histopathologically	68	26	22	136
Kim	2014	South Korea	Retro	252, 253	0, 750	1.5	0.986	0.91	1.27	Bilateral breast surface	Biopsy	69	26	22	136
Schipper	2014	Netherlands	Prosp	50, 135	0, 500 800	3.0	0.65	0.72	0.75	32-channel cardiac sensitivity encoding	Histopathologically	14	46	8	67
Yamaguchi	2014	Japan	NG	16, 52	0, 800	1.5	0.852	0.75	1.03	Breast	Biopsy	13	3	3	17
Razek	2015	Egypt	Prosp	34, 65	0, 500 1000	1.5	1.30	1.08	1.15	8-channel breast	Histopathologically	41	0	3	21

*tp* true positive, *fp* false positive, *fn* false negative, *tn* true negative, *NG* not given, *Prosp* prospective, *Retro* retrospective

**Table 2:** Results of the evaluation of each study according to QUADAS-2

	1	2	3	4	5	6	7	8	9	10	11	12	13	14	Score
Nakai	+	+	+	?	+	+	+	+	?	?	?	+	+	+	10
Kamitani	+	+	+	?	+	+	+	+	−	?	?	+	+	+	10
Fornasa	+	+	+	+	+	+	+	+	?	?	?	+	+	+	11
He	+	+	+	?	+	+	+	+	+	?	?	+	+	+	11
Luo	+	+	+	?	+	+	+	+	?	?	?	+	+	+	10
Chuang	+	+	+	?	+	+	+	+	+	+	?	+	+	+	12
Kim	+	+	+	+	+	+	+	+	?	+	?	+	+	+	12
Schipper	+	+	+	+	+	+	+	+	+	?	+	+	+	+	13
Yamaguchi	+	+	+	?	+	+	+	+	+	?	?	+	+	+	11
Razek	+	+	+	+	+	+	+	+	+	?	?	+	+	+	12

+ = no bias; − = potential bias; ? = bias unclear; 1 = representative spectrum?; 2 = selection criteria clearly described?; 3 = acceptable reference standard?; 4 = time interval between MRI and pathology?; 5 = partial verification avoided?; 6 = differential verification avoided?; 7 = incorporation avoided?; 8 = description execution of DW-MRI?; 9 = description execution of pathology?; 10 = pathology results blinded?; 11 = DW-MRI results blinded?; 12 = clinical data available as in practice?; 13 = uninterpretable results reported?; 14 = withdraw explained?

### Heterogeneity test

We evaluated heterogeneity through *I*^2^ index in Stata 12.0. We found *P* < 0.05 and *I*^2^ = 8.7 % indicating there was existing heterogeneity in the included studies. We used Spearman correlation coefficient in Meta-DiSc version 1.4 to check threshold effect. We found that the value of Spearman correlation coefficient was −0.064 and *P* = 0.852. We can confirm that threshold effect was not the source of heterogeneity in this meta-analysis.

In order to assess the source of heterogeneity further, we applied meta-regression (Table 3) and subgroup analysis by adding nation, design, *b* value, field strength, and coil (Table 4). Meta-regression and subgroup analysis showed that the source of heterogeneity cannot be found in these factors (*P* > 0.05). However, among these studies, the heterogeneity was highly significant (*I*^2^ > 50 %). Thus, we should interpret the results carefully.

**Table 3:** Result of meta-regression analysis

Variable	Coefficient	Standard error	*P* value	Diagnostic odds ratio	95 % CI
Nation	0.594	0.868	0.513	1.81	(0.24~13.41)
Field strength	−1.636	0.942	0.120	0.19	(0.02~1.71)
*b* value	−0.266	1.275	0.838	0.77	(0.04~13.15)
Design	0.165	1.141	0.888	1.18	(0.08~16.38)
Coil	−0.443	1.334	0.749	0.64	(0.03~15.07)

*CI* confidence interval

### Diagnostic performance of DWI and publication bias

**Fig. 2:**
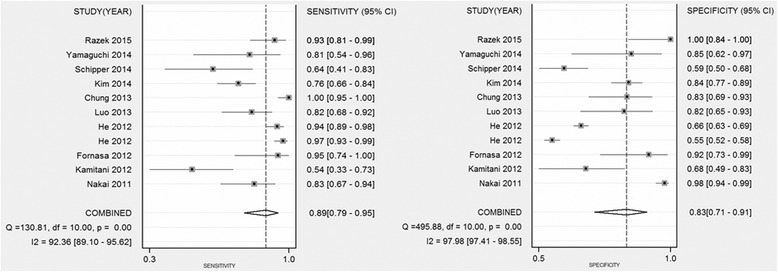
Forest plots of sensitivity and specificity with corresponding 95 % CIs of 18 studies. The pooled sensitivity and specificity were 0.89 (95 % CI 0.79–0.95) and 0.83 (95 % CI 0.71–0.91), respectively

**Fig. 3:**
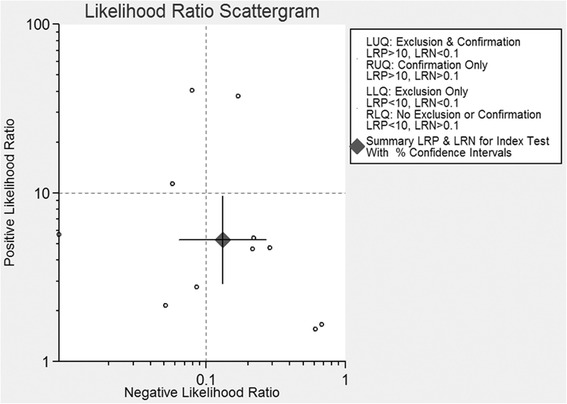
Scattergram of the PLR and NLR. Pooled estimates for the DWI were as follows: PLR was 3.86 (95 % CI 2.75–5.41), and NLR was 0.17 (95 % CI 0.09–0.32). *LLQ* left lower quadrant, *LRN* negative likelihood ratio, *LRP* positive likelihood ratio, *LUQ* left upper quadrant, *RLQ* right lower quadrant, *RUQ* right upper quadrant

**Fig. 4:**
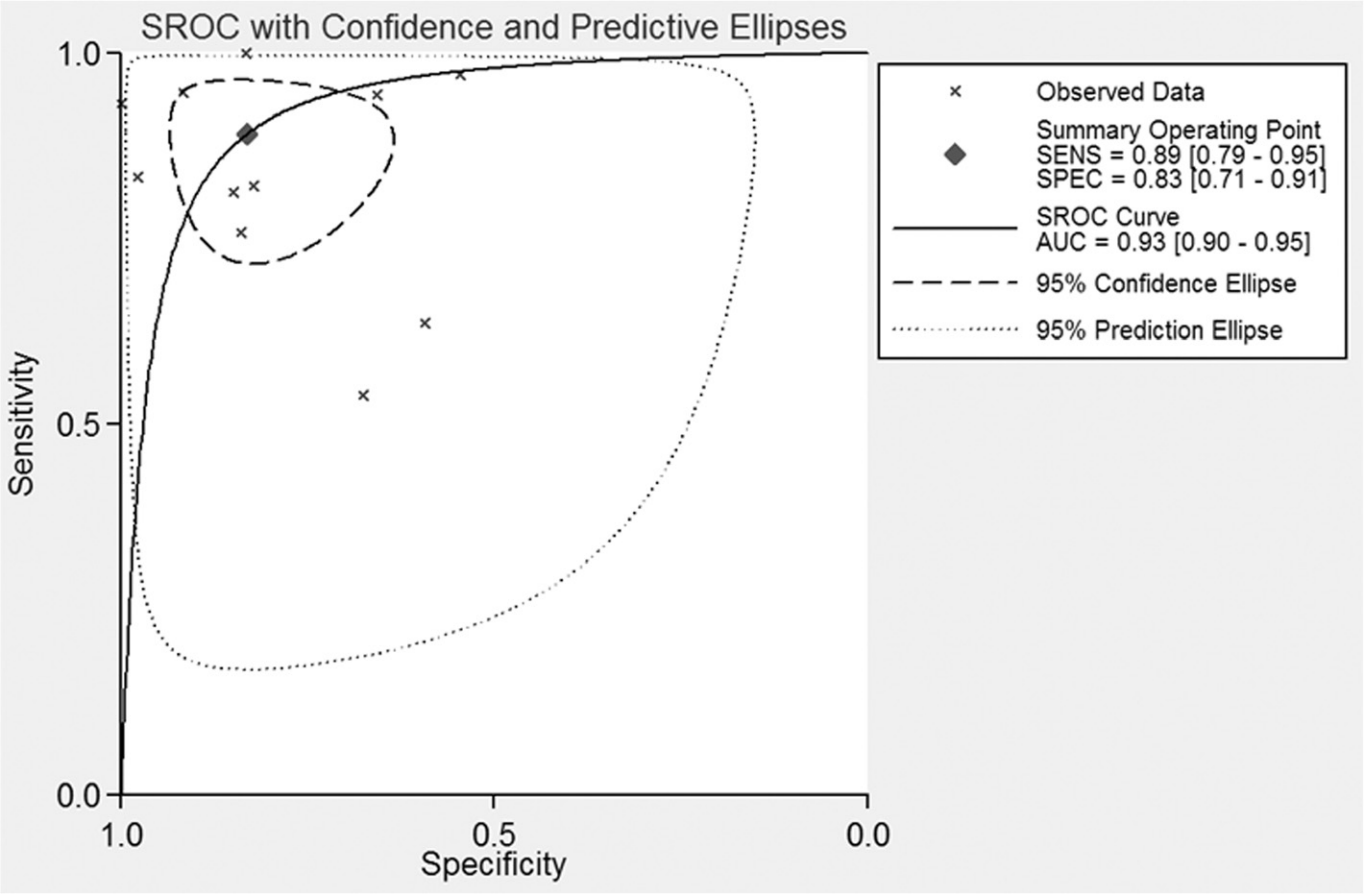
SROC curve for the diagnostic performance of DWI for all 10 studies combined. The pooled ROC with corresponding 95 % CI was 0.93 (95 % CI 0.90–0.95)

**Fig. 5:**
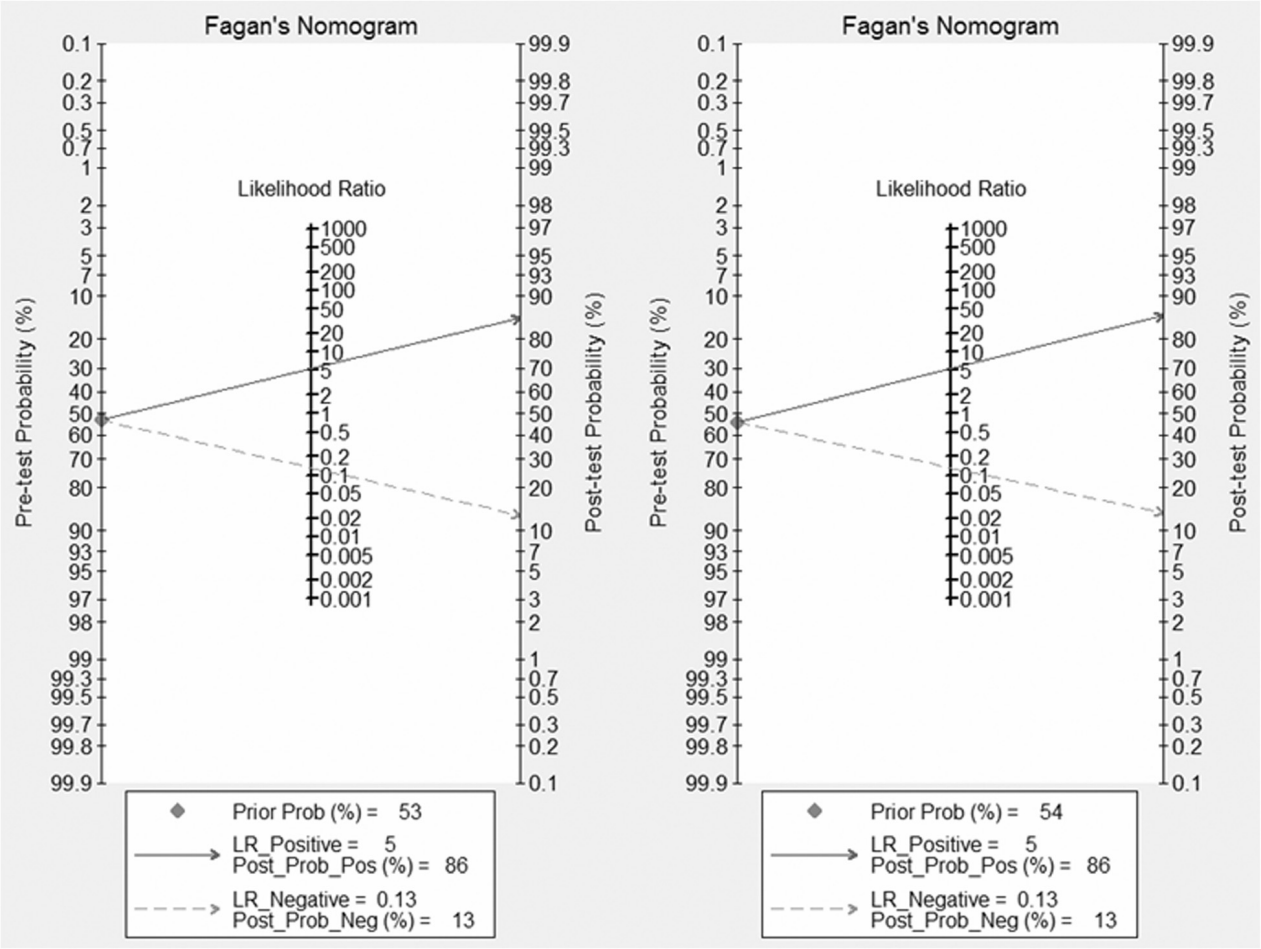
Posttest probabilities were calculated in Fagan’s nomograms by using different pretest probabilities of ALN metastases. The *left*, *middle*, and *right parts* represented the 53 and 54 % pretest probabilities

The sensitivity, specificity, PLR, NLR, and the AUC of DWI were 0.89 (95 % CI 0.79–0.95), 0.83 (95 % CI 0.71–0.91), 3.86 (95 % CI 2.75–5.41), 0.17 (95 % CI 0.09–0.32), and 0.93 (95 % CI 0.90–0.95), respectively (Figs. 2, 3, and 4). Figure 5 shows that the post probability positive (PPP) and post probability negative (PPN) were 86 and 13 %, respectively, when the pretest probabilities were defined as 53 and 54 %.

**Fig. 6:**
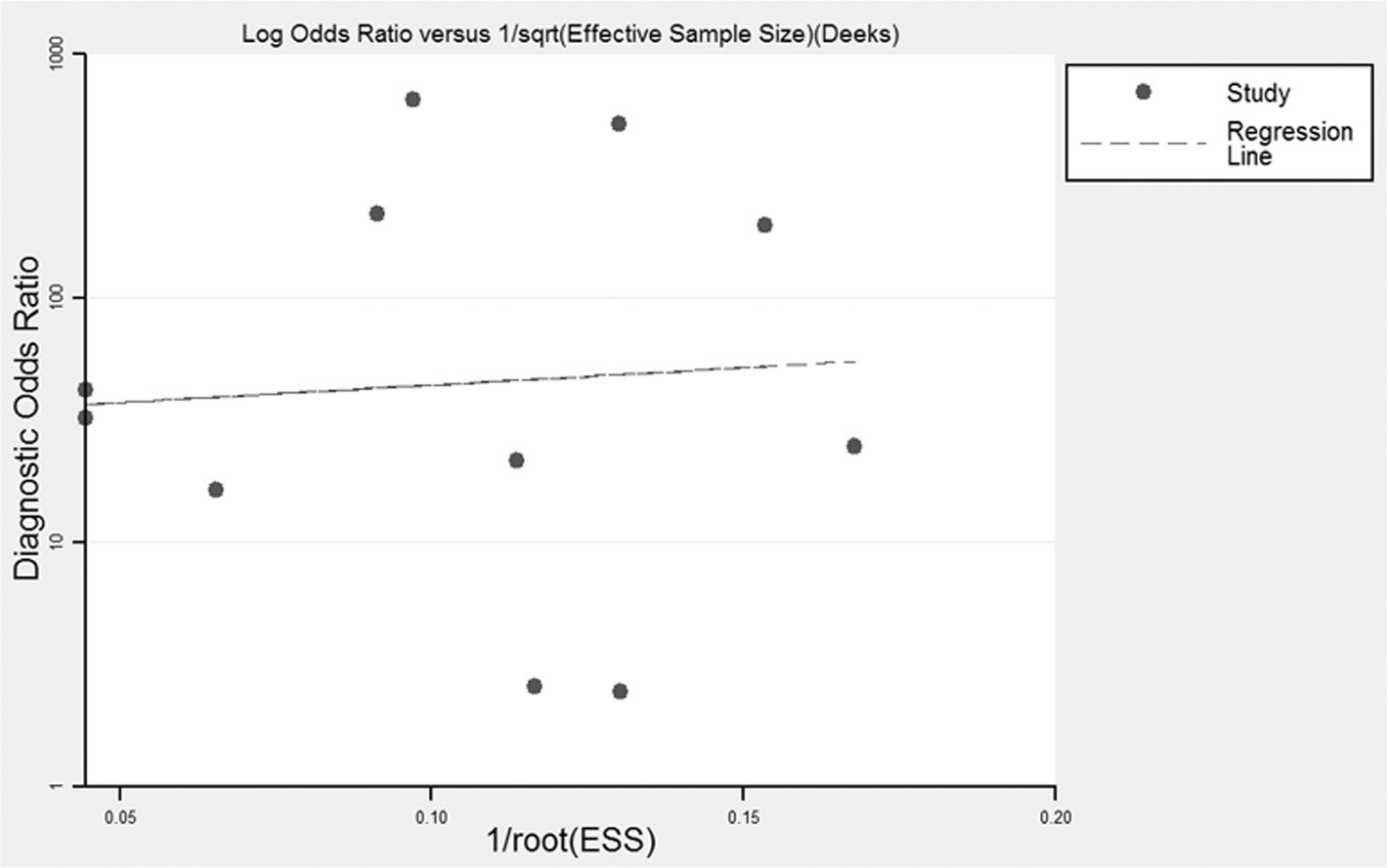
Deeks’ funnel plot indicated no publication bias (*P* = 0.759)

As presented in Fig. 6, Deeks’ funnel plot indicated that we found no significant publication bias among the studies (*P* = 0.759).

### The results of ADC values

**Fig. 7:**
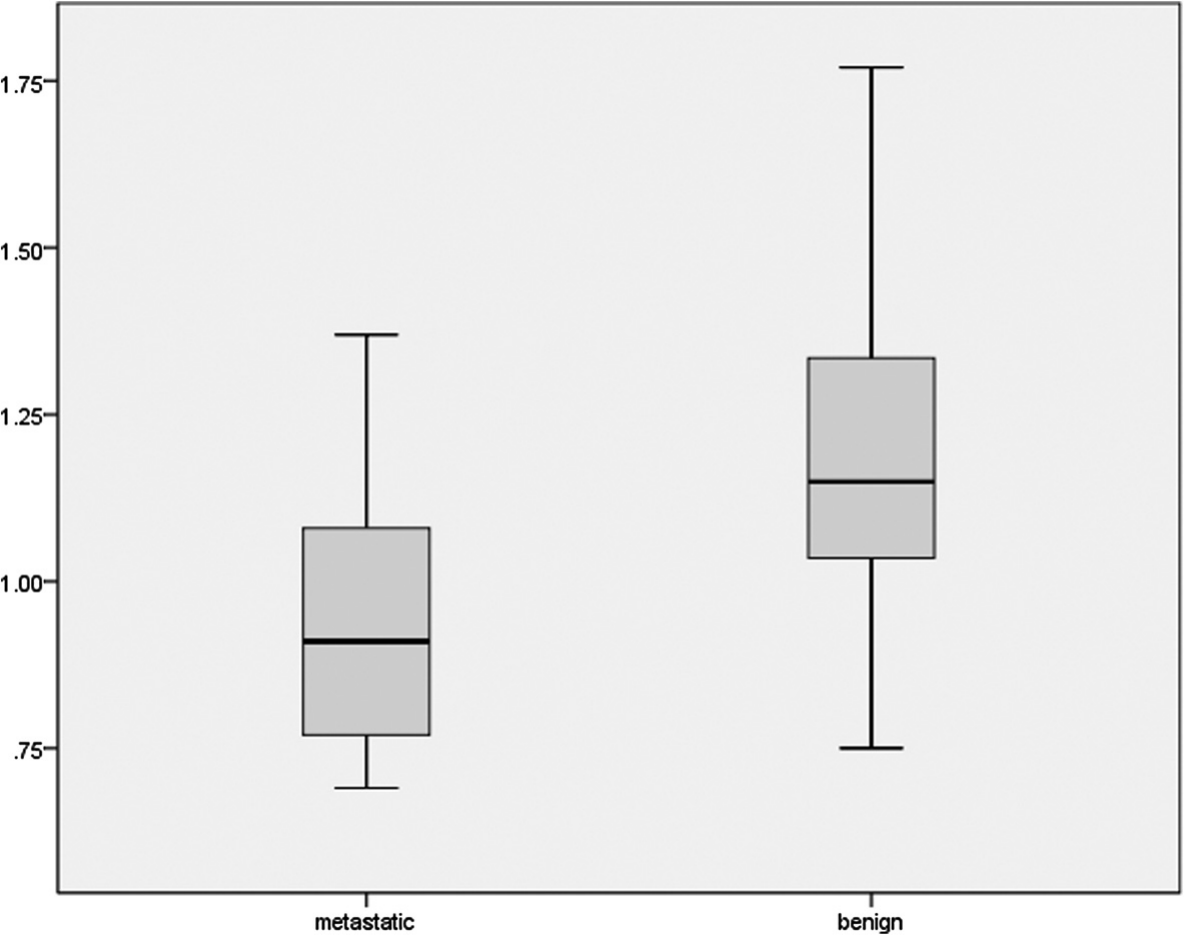
Box plot showed the comparison of the mean ADC value of metastatic and nonmetastatic ALNs. The mean ADC value of metastatic ALNs was significantly lower than that of the nonmetastatic ones (*P* < 0.000)

We compared the mean ADC values of metastatic and benign ALNs by Student’s *t* test. Figure 7 shows that the mean ADC value of metastatic ALNs was significantly lower than that of benign ALNs (*P* < 0.000).

## Discussion

Diffusion-weighted MR is a noninvasive technique to reflect functional information of tissues and is widely used in diagnosing the malignant lesions. Recently, detecting breast cancer and metastatic ALNs by DWI is a hot spot. We desire to assess the clinical significance of DWI in diagnosing metastatic ALNs in patients with breast cancer through systemic review. We selected 10 studies following the included criteria. We got the high sensitivity, specificity, and AUC as 89, 83, and 93 %, respectively. The results demonstrated that DWI was a promising method to differentiate diagnosis metastatic from nonmetastatic ALNs.

We also calculated the PLR and NLR to assess the performance of DWI. PLR and NLR are recognized as the better parameters to test the diagnostic accuracy. They can interpret one diagnostic tool through probability. We can rule in a disease when the PLR > 10 and rule out a disease when the NLR < 0.1 [26]. The PLR and NLR were 3.86 (95 % CI 2.75–5.41) and 0.17 (95 % CI 0.09–0.32), respectively. The results indicated that DWI could not rule in or rule out metastatic ALNs. Combined with the PLR and NLR, DWI had an inferior capacity to exclude or confirm metastatic ALNs. Fagan’s nomogram was also used to estimate the probability of having a disease in patients [27]. In this meta-analysis, the pretest probabilities of a patient with suspected metastatic ALNs were 53 and 54 %, and the posttest probability positive and posttest probability negative were 68 and 13 %, respectively, after likelihood ratio. The results meant that the pretest probabilities of 53 and 54 % were the cutoff for DWI-diagnosed metastatic ALNs. We can confirm the metastatic ALNs in patient when the pretest probabilities were higher than 53 and 54 % and excluded the metastatic ALNs in patient when the pretest probabilities were lower than 53 and 54 %. Therefore, the performance of DWI in differential diagnosis between metastatic and benign ALNs needs to be lucubrated.

ADC derived from DWI can determine the malignant lesions quantitatively and excluded the effect of T2 shine through [28]. Brownian motion of water molecules in cells can be quantitatively reflected. Wang et al. [29] found that the density of ALNs had significant positive correlation with metastatic lymph nodes and had significant negative correlation with ADC value. It meant that the diffusion of metastatic ALNs was higher than that of the benign ones and the ADC value of metastatic ALNs was lower than that of the benign ones. In Fig. 7, we found that the mean ADC value of metastatic ALNs was lower than that of the benign ones (*P* < 0.000). However, Xue et al. [30] and Roy et al. [31] got the opposite results. Actually, owing to the different selected ROI [32], liquefied active necrosis [33], and inflammatory and fibrous connective tissue proliferation [29], we acknowledged that certain overlap existed in metastatic and benign lymph nodes. In order to avoid the overlap, applying ratio of lymph node ADC value to primary tumor ADC value [34] and relative ADC values [35] may be a better method for detecting lymph node metastasis. Therefore, the clinical significance of different kinds of ADC values has more perspective. In this meta-analysis, we also found that the cutoff of diagnosing metastatic ALNs were variable and had no statistical significance.

Interestingly, in previous animal research [29], they found that the effect of perfusion can be ignored with *b* value ≥1000 and the diagnostic performance was better than that of the low *b* value. Previous study also demonstrated that with higher *b* value, the more accuracy the ADC had in other tumor [36]. Combined with professional knowledge, *b* value may be one of the sources of heterogeneity. In selected studies, the diagnosis performance with high *b* value was higher than with the lower ones (Table 4). However, with a low *b* value, owing to the perfusion effect, water diffusion motion cannot be reflected accurately. Moreover, the risk of distortion and susceptibility artifacts existing in DWI with high *b* value also cannot be ignored [37]. Different *b* values can affect the measurement of ADC and cutoff of ADC. It is necessary to get optimal *b* value for us to assess the statues of ALNs. Unifying the parameters of scan, cutoff of ADC, and optimal *b* value and improving spatial resolution were the direction of DWI in diagnosing ALN metastases in the future.

**Table 4:** The results of subgroup analysis

Variable	Number	Pooled sensitivity	*I* ^2^ (%)	Pooled specificity	*I* ^2^ (%)	Positive likelihood ratio	Negative likelihood ratio	Diagnostic odds ratio
Retrospective	3	0.729 (0.664–0.789)	60.2 %	0.846 (0.807–0.879)	0.0	4.628 (3.627–5.904)	0.350 (0.239–0.514)	14.358 (9.519–21.655)
Prospective	7	0.898 (0.860–0.929)	78.8 %	0.625 (0.600–0.650)	97.1	6.152 (2.387–15.857)	0.156 (0.072–0.342)	43.259 (10.752–174.0)
Field strength (1.5 T)	8	0.841 (0.806–0.872)	85.0 %	0.678 (0.655–0.699)	97.1	6.513 (2.884–14.711)	0.195 (0.12–0.314)	31.716 (15.746–63.884)
Field strength (3.0 T)	2	0.732 (0.640–0.811)	18.2 %	0.738 (0.682–0.789)	95.2	2.716 (0.874–8.438)	0.409 (0.197–0.849)	6.644 (1.086–40.634)
*b* value <1000 (s/mm^2^)	8	0.867 (0.831–0.899)	82.0 %	0.646 (0.6239–0.669)	97.1	5.801 (2.575–13.066)	0.177 (0.09–0.323)	35.007 (12.019–101.96)
*b* value ≥1000 (s/mm^2^)	2	0.769 (0.696–0.832)	86.8 %	0.861 (0.814–0.901)	71.1	4.888 (2.753–8.679)	0.264 (0.120–0.580)	19.496 (5.243–72.498)

We should acknowledge the limitations of this meta-analysis. First, with the limited number of selected studies, we cannot find the source of heterogeneity. Second, the potential publication bias could not be ignored, although our result showed no significant publication bias. Third, lacking enough high-quality prospective studies may uncover the ability of DWI detecting the metastatic ALNs.

In conclusion, DWI is a promising diagnostic method for differentiation between benign and metastatic ALNs. ADC value can quantitatively analyse ALNs. Larger number of high-quality prospective studies regarding DWI and ADC to detect ALN metastases still need to be performed.
